# Comparison of Nutrient Estimates Based on Food Volume versus Weight: Implications for Dietary Assessment Methods

**DOI:** 10.3390/nu10080973

**Published:** 2018-07-27

**Authors:** Emma K. Partridge, Marian L. Neuhouser, Kara Breymeyer, Jeannette M. Schenk

**Affiliations:** 1 Nutritional Sciences Program, University of Washington, Seattle, WA 98109-1024, USA; emma.k.partridge@uwalumni.com (E.K.P.); mneuhous@fredhutch.org (M.L.N.); 2 Cancer Prevention, Fred Hutch Cancer Research Center, Seattle, WA 98109, USA; kbreymey@fredhutch.org

**Keywords:** nutrition, food measurement, nutrient database, dietary assessment

## Abstract

Novel technology-based dietary assessment methods use volume estimates of foods to assess dietary intake. However, the nutrient content of standard databases is based on food weight. The goal of this study is to evaluate the accuracy of the United States Department of Agriculture National Nutrient Database for Standard Reference (USDA-SR) estimates of volume and the corresponding macronutrient content of the foods. The weights of 35 individual food volumes were measured (on trial) and compared to the USDA-SR-determined weight for the food volume. Macronutrient content corresponding to the trial weight and the USDA-SR weight for the food volume (USDA) were determined using the USDA-SR, and the differences were calculated. There were statistically significant differences between the USDA and trial weights for 80% of foods measured. Calorie estimates by USDA weight were significantly lower than that of trial weight for 54% of foods but were significantly greater for 26% of foods. Differences in macronutrient estimates by trial and USDA weight varied by food type. These findings suggest that nutrient databases based on food weight may not provide accurate estimates of dietary intake when assessed using food volumes. Further development of image-assisted dietary assessment methods which measure food volumes will necessitate evaluation of the accuracy of the processes used to convert weight to volume in nutrient databases.

## 1. Introduction

Traditional dietary assessment tools, such as multiple-day food records and interviewer-assisted 24-h recalls, rely on self-assessment of the amounts of foods eaten. However, it is a well-documented fact that people cannot accurately recall or estimate the amount of food they consume [1,2,3]. Emerging technology-based dietary assessment methods that use images to assess the types and amounts of foods people consume have the potential to provide more objective estimates of dietary intake. As part of the development and validation of these new methods, it will be important to consider the accuracy of standard nutrient databases to estimate nutrient content information from food volumes as opposed to weights. 

The United States Department of Agriculture National Nutrient Database for Standard Reference (USDA-SR) [4] is the primary source of food composition data in the US and serves as the foundation for most public and private food and nutrient databases, such as the Nutrition Data System for Research (NDS-R) [5]. USDA-SR is compiled of data from published and unpublished sources, including the Food and Nutrient Database for Dietary Studies (FNDDS), studies conducted by the USDA and contractors, lab analyses, algorithms, factors, or recipes [6]. The nutrient content information in these databases is largely based on food weights, not volumes, as food weight is considered the gold standard of measurement [6,7]. Although density factors have been developed for many of the foods to enable their conversion into household (volume) measures, the algorithms or processes used within USDA-SR are not fully known. 

In this study, we evaluate the extent to which estimates of food portion sizes measured by volume differ from those measured by weight, and assess the subsequent differences in estimated macronutrient content of these food portion sizes when based on volume and weight.

## 2. Materials and Methods 

### 2.1. Sample Size and Food Selection

Trials were performed on a total of 35 individual foods. Foods from each of the six USDA MyPlate [8] food categories (fruits, vegetables, grains, dairy, protein foods, and fats/oils) were selected to reflect the foods most commonly consumed by Americans, while allowing for variation in water content and shape [9,10]. Combination or mixed foods, such as soups and casseroles, were excluded for these experiments. A single preparation method was selected for most foods, and for a small number of foods (*n* = 4), multiple preparation methods were applied in order to assess differences in weight and nutrient content for one food prepared in multiple ways. Independent trials were completed on ten percent of foods chosen at random (potato (½ cup and 10 fries), chicken breast (whole and chopped), ice cream, regular salad dressing) for quality control. 

### 2.2. Trial Volumes

Selected trial volumes for most individual food trials were based on MyPlate portion sizes [8]. Fruits and vegetables were measured as ½ cup-equivalents, grains as two ounce-equivalents, and dairy as one-cup equivalents, except ice cream, which was measured according to the serving size portion listed on the Nutrition Facts panel (½ cup). Protein foods were measured as individual portion sizes (patty, breast, large egg) between one or three ounce-equivalents, except bacon (three slices), which was measured according to the serving size portion listed on the Nutrition Facts panel. Fats/oils were measured between one and two tablespoons, depending on the individual food [8]. 

### 2.3. Preparation Methods

All foods were prepared in a commercial-grade metabolic research kitchen at the Fred Hutch Cancer Research Center (FHCRC) Human Nutrition Lab (HNL) by a single trained dietetic technician (EKP). Foods needing no preparation, such as raspberries, were measured ‘as purchased’. If a food needed to be manipulated, alterations included first removing inedible portions, then size being manipulated (for example, sliced, chopped, or diced). Foods not commonly consumed raw were cooked according to protocols used by the FHCRC HNL, or by packaging instructions. The preparation method for each food was chosen based on available options in the USDA-SR 28 [4]. The LanguaL Thesaurus [11] was consulted to define standard size manipulations, and parchment paper with cut size markings was used for guidance. Details of selected foods, volumes, and preparation methods are available in Table 1. 

### 2.4. Data Collection 

For each trial, a prepared food was weighed at the test volume to determine its weight in grams, herein referred to as trial weight. Ten replicates per preparation method were completed for each food. Foods were measured at the test volume using standard food measuring tools; the same instrument was used throughout each trial and cleaned (washed and dried) between replicates. For each food trial, the USDA weight and macronutrient content for the test volume was determined by entering the test volume directly into USDA-SR to yield the corresponding gram weight and macronutrient content. For a small number of foods (*n* = 3), a volume option was not available in the USDA-SR; thus, the Nutrition Data System for Research (NDSR) database (University of Minnesota, Minneapolis, MN, USA) [5] was used to determine the corresponding gram weight and macronutrient content. 

### 2.5. Statistical Analysis 

For each food trial, the means and standard deviations of the 10 trial weight replicates were calculated. The USDA weight was set for each test volume at the value obtained from the USDA-SR database. Percentage differences between the trial and USDA weights, defined as the difference of trial weight subtracted from USDA weight divided by USDA weight, were determined for each replicate, and overall mean percentage differences between the trial and USDA weight were calculated. Similar methods were used to determine absolute differences in macronutrient content between trial and USDA weight for the selected trial volume of an individual food. For each food, one-sample *t*-tests were used to evaluate whether the mean differences between trial (average of 10 replicates), USDA weights, and nutrient contents were significantly different from zero. Statistical analyses were conducted using Statistics and Data (STATA) software (Release 14, College Station, TX, USA).

## 3. Results

Table 2 summarizes the mean trial weight, USDA weight, and mean percentage difference in trial and USDA weight for test volumes of individual foods. For 80% of food trials, there were statistically significant relative differences between the USDA and trial weights of the selected trial volume, ranging from −103.4% for sliced onions to +38.7% for shredded cheddar cheese. Within individual food groups, relative differences between USDA and trial weights were statistically significant for 65% of fruit and vegetable, 67% of grain, 100% of dairy, 77% of protein, and 100% of fat/oil foods, though there were no discernable patterns in either the direction or magnitude of relative weight differences across food categories.

Table 3 provides estimates of calorie and macronutrient content corresponding to the USDA and trial weights, and their differences, for selected volumes of individual foods. Absolute differences between USDA and mean trial weight-derived calorie estimates for selected food volumes ranged from 0 to 60 kcal, and largely mirrored those reported for weights (Table 2). For 52% of food trials, calories determined by USDA weight were significantly lower than by trial weight, and for 26% of foods, calories determined by USDA were significantly greater than by trial weight, although the absolute value of these differences was small for many foods. The largest calorie differences between USDA and trial weight were found for dairy foods; calories determined by trial weight for ice cream were 60 ± 3 kcal less than by USDA weight (*p* < 0.0001). Conversely, for shredded cheddar cheese, calories determined by trial weight were 59 ± 2 kcal greater than by USDA weight (*p* < 0.0001). 

Differences in estimated macronutrient content between USDA and trial weight were dependent on food type (Table 3). Higher-fat foods, like shredded cheddar cheese and nuts, tended to have the largest absolute differences in estimates of fat content, although the direction of differences was inconsistent. In general, the absolute differences in estimated protein content were small for fruit and vegetable foods, which have lower protein content, but were quite large for protein and dairy foods. Similarly, absolute differences in estimated carbohydrate content between USDA and trial weight were largest for grains, dairy, and vegetables, but were relatively small for protein foods and fats/oils (Table 3).

## 4. Discussion

In this study we compared weights for selected food volumes measured in a research kitchen with those derived from the USDA-SR database. Overall, we found statistically significant differences between the USDA-derived and trial weights for 76% of the foods tested. In addition, there were significant differences in corresponding calorie estimates derived from the USDA and trial weights for 78% of foods. These findings suggest that the processes used to convert weight into volume in the USDA-SR may not provide accurate estimates of volume for many foods and may subsequently lead to inaccurate estimates of caloric and nutrient intake. 

Efforts to develop improved methods of dietary assessment that employ more objective measures of intake have recently gained attention [12,13,14], with many innovative technologies focusing on the use of images to estimate food volumes [15,16,17,18,19,20]. Using images to calculate volumes, these methods hold promise to provide more accurate estimates of the amounts of foods which people eat. The potential of these novel approaches, however, may be limited by the fact that nutrient content information in available databases is currently based on food weights, and estimates of food density, or weight for unit food volume, are required to convert volume into weight. For many foods in these databases, food density has been generated; however, little information is available about the algorithms and processes used to convert weight to volume, and the accuracy of these data are uncertain [6,21,22].

For most of the foods evaluated in this study, USDA weights for the selected trial volume tended to be greater than the measured weights. For some foods—primarily, fruits, vegetables, and fats/oils—the absolute differences between the USDA-derived and trial weights for food volume were modest, indicating that the algorithms or processes used to convert weight to volume for these foods were relatively accurate. For other foods, such as dairy, high-protein, and some manipulated or prepared foods, there were substantial differences between the measured and USDA weights for the trial food volume. Differences between the USDA and measured weight for a given volume may, in part, have been due to the protocols followed for manipulating or preparing foods. For example, many foods that required cooking preparations, such as potato, chicken, egg, bacon, and rice, had statistically significant differences between the USDA and trial weights (all *p* < 0.05). Cooking time, heat intensity, and water retention/release can vary through the cooking process and between protocols used (trial vs. USDA), which may impact cooking yield, thereby contributing to the observed differences in USDA and trial weight for the selected test volume. For foods that were manipulated, variations in packing and differences in protocols used for manipulation may account for some differences between the USDA and measured weight for a given food volume. In our trials, manipulation methods were defined by the LanguaL Thesaurus and were standardized across trials. For some foods in the USDA-SR, different manipulation methods of the same food are grouped together, such as quartered and chopped apples. As a result, the weight and nutrient content for the same volume of each form of the food is identical, even though the size, shape, and air space upon packing differs greatly. It is important to note that image-assessed food volumes will inherently include air space, due to food packing; therefore, nutrient database conversions for weight to volume will need to be equivalently determined.

Because weight is the standard by which USDA-SR determines nutrient content, differences between the USDA-derived and trial weight for a given food volume yielded corresponding differences in calorie and macronutrient content estimates. Over 70% of foods, regardless of food group, that had significant differences between USDA-derived and trial weight for a given food volume also had corresponding significant differences in calorie estimates, though we found no apparent pattern within or across food groups in the magnitude or direction of these differences. However, the corresponding differences between USDA and trial weight-derived macronutrient content was dependent on the nutrient composition of the individual food; foods dense in a specific macronutrient tended to have greater differences in that macronutrient. For example, for high-fat foods such as pecans, even small absolute differences between the USDA-derived and trial weights yielded substantial differences in calorie and fat content estimates (difference between USDA–trial weight-derived weight, calories and fat: −7.8 g, −53.7 kcal; −5.6 g fat (both *p* < −0.0001)). The overall impact of differences between weight and volume-based measures of dietary intake will depend heavily on the individual foods people eat. In order to further evaluate this potential impact, the extent to which estimates of food portion sizes measured by volume differ from those measured by weight would need to be measured for an extensive list of foods.

To our knowledge, this is the first study to report differences in nutrient database information by volume and weight. Foods were systematically selected based on popularity in the US diet, and were measured and prepared via standardized methods defined by the LanguaL Thesaurus. In addition, multiple replicates were measured for each food and food preparation method, to align with the sampling methods used for USDA-SR. However, this study is not without limitations. Data from individual replicates were not publicly available for USDA-SR; thus, our estimates of mean differences between the trial and USDA relied only on a single value of the USDA mean, which may be anti-conservative. For some foods, the number of replicates used in this study (*n* = 10) may be less than that assessed by the USDA, which would reduce the accuracy of our measurements compared to those made by the USDA. In addition, while most preparation methods were available via the LanguaL Thesaurus, cooking heat and time were unavailable for most cooked foods. Instead, protocols defined by the Human Nutrition Laboratory at the Fred Hutchinson Cancer Research Center were followed, and may differ from those used by the USDA. For our trials, we purposely selected generic options; thus for some foods, the differences reported here may also reflect differences in the exact foods measured. Lastly, this study was limited to single and non-mixed foods. Although the foods chosen represent foods commonly eaten by Americans, they may be less representative of foods prevalent in everyday diets.

## 5. Conclusions

This study demonstrates that for selected food volumes, substantial differences existed between the corresponding USDA-derived and trial weights measured in a research kitchen. The differences between the USDA-derived and trial weights for a selected food volume also resulted in parallel differences between estimated macronutrient content. As the primary source of food composition data in the US, researchers rely heavily on the USDA-SR database, either directly or indirectly, to estimate dietary intake of nutrients. 

Given the development of new image-assisted dietary assessment methods that provide objective measures food volume, it is important to assess the accuracy of nutrient databases to estimate nutrient content based on food volumes. The findings reported here suggest that the estimation of dietary intake using food volumes may not provide accurate estimates of nutrient intake. Most nutrient databases commonly used in the US are based on USDA-SR nutrient data, and thus would be affected by the same inaccuracies. Whether the same issues are apparent in other food and nutrient databases around the world is unknown. In order to better understand the impact of these discrepancies on assessment of dietary and micronutrient intake, further evaluation of the accuracy of processes used to convert weight to volume in the USDA-SR is warranted. 

## Figures and Tables

**Table 1:** Descriptions of selected foods and preparation methods used for trials.

		Measured Portion Sizes	Preparation Method		As Purchased or Edible Portion	USDA Food Description	Purchased Food Description (If Applicable)	USDA Number
**Fruits**								
Strawberries		½ cup	Halves		Edible Portion	Strawberries, raw		09316
Cantaloupe		½ cup	Cubed ^2^		Edible Portion	Melons, cantaloupe, raw		09181
Peaches		½ cup	Sliced ^2^		Drained of Liquids	Peaches, canned, heavy syrup, drained		09370
Oranges		½ cup	Sectioned, with and without membranes		Edible Portion	Oranges, raw, all commercial varieties	Navel Oranges	09200
Raspberries		½ cup	Whole		As Purchased	Raspberries, raw		09302
Apples		½ cup	Quartered, sliced, and chopped ^2^		Edible Portion	Apples, raw, with skin	Gala Apples	09003
Seedless Grapes		½ cup	Whole		Edible Portion	Grapes, red or green (European type, such as Thompson seedless), raw	Red Grapes	09132
Bananas		½ cup	Sliced ^2^		Edible Portion	Bananas, raw		09040
Avocados		½ cup	Cubed ^2^		Edible Portion	Avocados, raw, all commercial varieties		09037
Raisins		¼ cup ^1^	Packed		As Purchased	Raisins, seedless	Generic brand (Kroger), seedless from green grapes	09298
**Vegetables**								
Iceberg Lettuce		½ cup	Chopped (loosely packed)		Edible Portion	Lettuce, iceberg (includes crisphead types), raw		11252
Tomatoes		½ cup	Chopped ^2^		Edible Portion	Tomatoes, red, ripe, raw, year round average		11529
Potatoes, French fries ^3^		½ cup & 10 fries ^1^	Oven-heated		As purchased, heated	Potatoes, French fried, crinkle or regular cut, salt added in processing, frozen, oven-heated	Ore Ida frozen French-fried potatoes, regular cut	11360
Onions		½ cup	Sliced and chopped ^2^		Edible Portion	Onions, raw	Yellow onions	11282
Sweet Corn		½ cup	Drained of liquids		As Prepared	Corn, sweet, yellow, canned, vacuum pack, regular pack	Santiam golden sweet whole kernel corn, canned	11176
**Grains**								
Bread		2 slices	Pre-Sliced		As Purchased	Bread, wheat	Generic brand (Kroger), wheat bread	18064
Pasta		1 cup	Boiled, not packed		As Purchased	Pasta, cooked, enriched, without added salt	Generic brand (Kroger), enriched spaghetti	20121
Rice		1 cup	Boiled		As Purchased	Rice, white, long-grain, regular, enriched, cooked	Generic brand (Kroger), long grain	20045
**Dairy**								
Cheese		⅓ cup	Shredded		As Purchased	Cheese, cheddar	Generic brand (Kroger), all natural medium cheddar—purchased in a block and grated	01009
Ice Cream ^3^		½ cup	None		As Purchased	Ice creams, vanilla	Generic brand (Kroger), Vanilla	19095
Yogurt		1 cup	None		As Purchased	Yogurt, plain, skim milk, 13 grams protein per 8 ounce	Generic brand (Fred Meyer), non-fat, plain	01118
**Protein**								
Beef, ground	7% fat	4” diameter patty	Pan-broiled		As Purchased	Beef, ground, 93% lean meat/7% fat, patty, cooked, pan-broiled	Generic brand (Kroger), 4” diameter using 114 g (¼ pound) of raw meat (patties were formed raw then cooked per HNL protocols)	23474
	30% fat	4” diameter patty	Pan-broiled		As Purchased	Beef, ground, 70% lean meat/30% fat, patty cooked, pan-broiled	Fresh from butcher (Marketime foods)	13496
Chicken Breast, without skin ^3^		Roasted	1 breast	Roasted, whole and chopped ^2^	As Purchased	Chicken, broilers or fryers, breast, meat only, cooked, roasted	Foster Farms, breast fillets	05064
Egg, large		Scrambled	1 large Egg	Scrambled	Edible Portion	Egg, whole, cooked, scrambled	Generic brand (Fred Meyer) large eggs, grade AA ^3^	01132
Bacon		Pan-fried	3 slices	Pan-fried	As Purchased	Pork, cured, bacon, pre-sliced, cooked, pan-fried	Generic brand (Fred Meyer), traditional cut, sugar cured	10862
Cashews		⅛ cup	Raw, whole		As Purchased	Nuts, cashew nuts, raw	Raw, whole, purchased in bulk	12087
Almonds		⅛ cup	Raw, whole		As Purchased	Nuts, almonds	Blue Diamond, raw, whole	12061
Pecans		⅛ cup	Raw, halves		As Purchased	Nuts, pecans	Diamond of California, raw	12142
Peanut Butter		2 tablespoons	None		As Purchased	Peanut butter, smooth style, with salt	Generic brand (Fred Meyer)	16098
**Fats, Oils**								
Margarine		1 tablespoon	None		As Purchased	Margarine, regular, hard, soybean (hydrogenated)	Generic brand (Fred Meyer)	04073
Canola Oil		2 tablespoons	None		As Purchased	Oil, canola	Generic brand (Kroger)	04582
Mayonnaise		2 tablespoons	None		As Purchased	Salad dressing, mayonnaise, regular	Generic brand (Fred Meyer)	04025
Dressing, Italian ^3^		2 tablespoons	None		As Purchased	Salad dressing, Italian dressing, commercial, regular	Generic brand (Fred Meyer), Zesty Italian	04114
Salsa		2 tablespoons	None		As Purchased	Sauce, salsa, ready-to-serve	Pace brand, Picante Medium	06164
Ketchup		2 tablespoons	None		As Purchased	Catsup	Generic brand (Fred Meyer)	11935

^1^ MyPlate volumetric equivalents. ½ cup fruit equivalent = ½ cup hydrated fruit = ¼ cup dried fruit. ½ cup vegetable equivalent = 10 French fries. ^2^ Standard preparation methods and their parameters are as follows: Sliced: between 0.5 cm and 1.5 cm [11]. For consistency, the investigator measured slices to 1 cm. Cubed: >1.5 cm. For consistency, the investigator attempted to cut each cube side to 2 cm. Chopped: Item divided into pieces with a thickness <0.3 cm [11]. For consistency, the investigator chopped items as close to 0.25 cm as possible. ^3^ Foods that were re-measured for quality control tests. ^4^ Grade AA eggs have thick, firm whites and high, round yolks. USDA: United States Department of Agriculture National Nutrient Database; HNL: Human Nutrition Lab.

**Table 2:** Comparison of mean measured (trial) weights of individual foods to the USDA-SR (United States Department of Agriculture National Nutrient Database for Standard Reference) 28 database weight for selected food volumes.

		Selected Test Volume	Trial Weight (g) Mean (sd) ^1^	USDA Weight (g) Mean ^2^	% Difference ^3^ Mean (se) ^4^
**Fruits**					
Strawberries, halved		½ cup	75.8 (5.5)	76.0	0.3 (2.3)
Cantaloupe, cubed		½ cup	85.7 (8)	80.0	−7.2 (3.2) ^6^
Peaches, sliced		½ cup	110.6 (4.4)	111.0	0.4 (1.3)
Orange sections	Membrane	½ cup	98.9 (5.1)	90.0	−9.9 (2.0) ^8^
	No Membrane	½ cup	98.9 (3.9)	90.0	−9.9 (1.4) ^8^
Raspberries, whole		½ cup	61.8 (3.8)	61.5	−0.5 (2.0)
Apples	Quartered	½ cup	76.7 (5.1)	62.5	−22.6 (2.6) ^8^
	Sliced	½ cup	87.3 (5.2)	54.5	−60.2 (3.0) ^8^
	Chopped	½ cup	67.31 (2.5)	62.5	−7.7 (1.3) ^7^
Grapes, seedless, whole		½ cup	91.8 (2.8)	75.5	−21.6 (1.2) ^8^
Bananas, sliced		½ cup	76.7 (4.5)	75.0	−2.3 (1.9)
Avocados, cubed		½ cup	90.0 (7.3)	75.0	−20.0 (3.1) ^8^
Raisins, packed		½ cup	44.2 (2.2)	41.25	−7.1 (1.7) ^7^
**Vegetables**					
Iceberg Lettuce, chopped		½ cup	30.0 (2.9)	28.5	−5.3 (3.2)
Tomatoes, chopped		½ cup	107.1 (4.1)	90.0	−19.0 (1.4) ^8^
Potatoes, French fries		10 fries	47.9 (6.8)	69.0	30.6 (2.2) ^8^
		½ cup ^5^	51.6 (3.6)	52.0	0.9 (1.6)
Onions, raw	Sliced	½ cup	116.9 (15.5)	57.5	−103.4 (8.5) ^8^
	Chopped	½ cup	81.3 (3.9)	80.0	−1.6 (1.5)
Sweet Corn, canned, drained		½ cup	87.5 (5.7)	105.0	16.6 (1.7) ^8^
**Grains**					
Bread, wheat, sliced		2 slices	58.4 (2.7)	58.0	−0.6 (1.4)
Pasta, enriched, spaghetti		1 cup	119.0 (3.4)	124.0	4.0 (0.9) ^7^
Rice, white, long grain, enriched		1 cup	182.7 (17.7)	158.0	−15.6 (3.5) ^7^
**Dairy**					
Cheddar cheese, shredded		⅓ cup	23.1 (1.5)	37.6	38.7 (1.2) ^8^
Ice Cream, vanilla		½ cup	95.3 (6.5)	66.0	−44.4 (1.5) ^8^
Yogurt, skim		1 cup	260.8 (1.2)	245.0	−6.4 (0.2) ^8^
**Protein**					
Beef, patty	7% fat	4” patty ^5^	84.2 (3.2)	81.6	−3.1 (1.2) ^6^
	30% fat	4” patty	71.2 (3.4)	77.0	7.5 (1.4) ^8^
Chicken Breast, without skin, roasted	Whole	1 breast	206.2 (43.8)	172.0	−19.9 (5.7) ^7^
	Chopped	1 cup	139.1 (3.9)	140.0	0.7 (0.7)
Egg, large, scrambled		1 large egg	43.9 (2.5)	61.0	28.0 (1.3) ^8^
Bacon, regular cut, pan-fried		3 slices	26.9 (5.3)	34.5	22.1 (4.8) ^7^
Cashews, raw, whole		⅛ cup ^5^	24.3 (1.9)	16.1	−50.7 (3.8) ^8^
Almonds, raw, whole		⅛ cup	24.8 (1.9)	17.9	−38.7 (3.3) ^8^
Pecans, raw, halved		⅛ cup	20.2 (1.3)	12.4	−63.2 (3.3) ^8^
Peanut Butter, smooth		2 T	29.7 (0.9)	32.0	7.2 (0.9) ^8^
**Fats and Oils**					
Margarine		1 T	14.3 (0.2)	14.1	−1.6 (0.5) ^6^
Canola Oil		2 T	25.5 (0.3)	28.0	8.8 (0.4) ^8^
Mayonnaise		2 T	29.0 (0.4)	27.6	−5.1 (0.4) ^8^
Dressing, Italian, regular		2 T	30.6 (0.9)	29.4	−4.0 (0.7) ^8^
Salsa		2 T	32.9 (1.5)	36.0	8.8 (1.3) ^8^
Ketchup		2 T	34.3 (0.3)	34.0	−1.0 (0.2) ^7^

^1^ Mean and standard deviation determined by 10 experimental weight replicates. ^2^ Estimate of variance not available from USDA-SR 28. ^3^ (USDA Weight—Trial Weight)/USDA weight. ^4^ Standard error from one-sample *t*-test. ^5^ Nutrient content unavailable in USDA-SR 28, value obtained from NDSR (Nutrition Data System for Research). ^6^ *p* < 0.05, ^7^ *p* < 0.01, ^8^ *p* < 0.001.

**Table 3:** Mean macronutrient values obtained from USDA-SR 28 based on trial volume and USDA volume and their differences (USDA Volume–Trial Volume).

		Calories (kcal)			Total Fat (g)			Protein (g)			Carbohydrate (g)		
		Weight		Diff: Mean (se)	Weight		Diff: Mean (se)	Weight		Diff: Mean (se)	Weight		Diff: Mean (se)
		Trial	USDA		Trial	USDA		Trial	USDA		Trial	USDA	
**Fruits**													
Strawberries, halved		24	24	−0.3 (0.56)	0.2	0.2	0 (0.01)	0.5	0.5	0 (0.01)	5.8	5.8	0.02 (0.13)
Cantaloupe, cubed		29	27	−2.3 (0.9) *	0.2	0.2	−0.01 (0) *	0.7	0.7	−0.05 (0.02) *	7	6.5	−0.47 (0.21) *
Peaches, sliced		80	80	0.4 (0.99)	0.2	0.2	0 (0)	0.6	0.6	0 (0.01)	20	20	0.08 (0.26)
Orange sections	Membrane	46	42	−4.4 (0.76) ***	0.1	0.1	−0.01 (0) **	0.9	0.9	−0.08 (0.02) ***	12	11	−1.04 (0.19) ***
	No Membrane	47	42	−4.5 (0.56) ***	0.1	0.1	−0.01 (0) ***	0.9	0.9	−0.08 (0.01) ***	12	11	−1.04 (0.15) ***
Raspberries, whole		32	32	0 (0.67)	0.4	0.4	0 (0)	0.7	0.7	0 (0.01)	7.4	7.3	−0.04 (0.14)
Apples	Quartered	40	32	−7.9 (0.81) ***	0.1	0.1	−0.02 (0) ***	0.2	0.2	−0.04 (0) ***	11	8.6	−1.96 (0.22) ***
	Sliced	45	28	−17.4 (0.87) ***	0.2	0.1	−0.06 (0) ***	0.3	0.1	−0.09 (0) ***	12	7.5	−4.53 (0.23) ***
	Chopped	35	32	−3 (0.37) ***	0.1	0.1	−0.01 (0) *	0.2	0.2	−0.01 (0) ***	9.3	8.6	−0.67 (0.11) ***
Grapes, seedless, whole		63	69	5.7 (0.58) ***	0.2	0.2	0.01 (0) ***	0.7	0.7	0.06 (0.01) ***	17	18	1.48 (0.16) ***
Bananas, sliced		68	67	−1.3 (1.26)	0.3	0.3	0 (0)	0.9	0.8	−0.02 (0.02)	18	17	−0.39 (0.32)
Avocados, cubed		144	120	−24.1 (3.73) ***	13	0.1	−2.19 (0.34) ***	1.8	1.5	−0.3 (0.05) ***	7.7	6.4	−1.28 (0.2) ***
Raisins, packed		132	123	−9.1 (2.09) **	0.2	0.2	−0.02 (0) **	1.4	1.3	−0.09 (0.02) **	35	33	−2.3 (0.56) **
**Vegetables**													
Iceberg Lettuce, chopped		4	4	−0.1 (0.1)	0	0	0 (0)	0.3	0.3	−0.01 (0.01)	0.9	0.9	−0.04 (0.03)
Tomatoes, chopped		19	16	−3.3 (0.26) ***	0.2	0.2	−0.03 (0) ***	0.9	0.8	−0.15 (0.01) ***	4.2	3.5	−0.67 (0.05) ***
Potatoes, French fries	10 fries	79	115	35.6 (2.5) ***	2.5	3.5	1.08 (0.08) ***	1.2	1.7	0.53 (0.04) ***	13	19	5.82 (0.42) ***
	½ cup	86	85	−0.55 (1.35)	2.7	2.7	0.07 (0.04)	1.3	1.2	−0.06 (0.02) **	14	14	0.25 (0.22)
Onions, raw	Sliced	47	23	−23.7 (1.94) ***	0.1	0.1	−0.06 (0) ***	1.3	0.6	−0.66 (0.05) ***	11	5.4	−5.55 (0.46) ***
	Chopped	32	32	−0.4 (0.52)	0.1	0.1	0 (0)	0.9	0.9	−0.01 (0.01)	7.6	7.5	−0.12 (0.12)
Sweet Corn, canned, drained		69	83	13.9 (1.39) ***	0.4	0.5	0.08 (0.01) ***	2.1	2.5	0.42 (0.04) ***	17	20	3.39 (0.35) ***
**Grains**													
Bread, wheat, sliced		156	155	−0.7 (2.19)	1.9	1.9	−0.01 (0.03)	6.3	6.2	−0.04 (0.09)	28	28	−0.18 (0.41)
Pasta, enriched, spaghetti		188	196	7.8 (1.7) *	1.1	1.2	0.04 (0.01) **	6.9	7.2	0.29 (0.06) **	37	38	1.53 (0.33) **
Rice, white, long grain, enriched		246	205	−40.5 (2.24) ***	0.5	0.4	−0.09 (0) ***	5.1	4.3	−0.83 (0.05) ***	53	45	−8.7 (0.48) ***
**Dairy**													
Cheddar cheese, shredded		93	152	58.8 (1.9) ***	7.7	13	4.85 (0.16) ***	5.3	8.6	3.34 (0.11) ***	0.7	1.2	0.45 (0.01) ***
Ice Cream, vanilla		197	137	−60.25 (3.02) ***	10	7.3	−3.23 (0.16) ***	3.3	2.3	−1.03 (0.05) ***	23	16	−6.92 (0.35) ***
Yogurt, skim		146	137	−9.2 (0.2) ***	0.5	0.4	−0.03	15	14	−0.9 (0.02) ***	20	19	−1.21 (0.03) ***
**Protein**													
Beef, patty ^a^	7% fat	153	155	1.8 (1.83)	6.7	6.8	0.01 (0.08)	22	22	0.56 (0.26)	0.1	0	−0.05 (0)
	30% fat	170	183	13.5 (2.53) ***	11	12	0.9 (0.17) ***	16	18	1.32 (0.24) ***	NA	NA	NA
Chicken Breast, without skin, roasted	Whole	340	284	−56.3 (16.14) **	7.4	6.1	−1.22 (0.35) **	64	53	−10.61 (3.03) **	NA	NA	NA
	Chopped	230	231	1.3 (1.45)	5	5	0.03 (0.03)	43	43	0.28 (0.27)	NA	NA	NA
Egg, large, scrambled		65	91	25.8 (1.15) ***	4.8	6.7	1.88 (0.09) ***	4.4	6.1	1.7 (0.08) ***	0.7	1	0.27 (0.01) ***
Bacon, regular cut, pan-fried		126	161	35.2 (7.83) **	9.4	12	2.68 (0.58) **	9.1	12	2.58 (0.56) **	0.5	0.6	0.13 (0.03) **
Cashews, raw, whole		134	89	−45.4 (3.35) ***	11	7.1	−3.58 (0.27) ***	4.4	2.9	−1.49 (0.11) ***	7.3	4.9	−2.47 (0.19) ***
Almonds, raw, whole		144	103	−40.5 (3.39) ***	12	8.9	−3.46 (0.29) ***	5.2	3.8	−1.47 (0.12) ***	5.4	3.9	−1.5 (0.13) ***
Pecans, raw, halved		140	86	−53.7 (2.8) ***	15	8.9	−5.64 (0.3) ***	1.9	1.1	−0.72 (0.04) ***	2.8	1.7	−1.08 (0.06) ***
Peanut Butter, smooth		178	191	13.3 (1.8) ***	15	16	1.19 (0.15) ***	6.6	7.1	0.52 (0.07) ***	6.6	7.1	0.52 (0.07) ***
**Fats and Oils**													
Margarine		103	101	−2.1 (0.5) **	12	11	−0.18 (0.06) *	0.1	0.1	0 (0.00)	0.1	0.1	0 (0.00)
Canola Oil		226	248	22.1 (0.85) ***	26	28	2.46 (0.10) ***	0	0	0 (0.00)	NA	NA	NA
Mayonnaise		197	188	−9.4 (0.79) ***	22	21	−1.05 (0.08) ***	0.3	0.3	−0.02 (0) ***	0.2	0.2	−0.01 (0) *
Dressing, Italian, Regular		73	71	−2.4 (0.5) ***	6.5	6.2	−0.25 (0.04) ***	0.1	0.1	−0.01 (0) ***	3.7	3.6	−0.15 (0.02) ***
Salsa		10	10	0.5 (0.17) *	0.1	0.1	0.003 (0)	0.5	0.6	0.05 (0.01) ***	2.2	2.4	0.21 (0.03) ***
Ketchup		35	34	−0.7 (0.15) **	0	0	0 (0.00)	0.4	0.4	−0.01 (0) **	9.4	9.3	−0.09 (0.02) **

NA: Not Applicable; ^a^ Nutrient content unavailable in USDA-SR 28, value obtained from NDSR. * *p* < 0.05, ** *p* < 0.01, *** *p* < 0.001.
